# Patient-Derived Cancer-Associated Fibroblasts Support the Colonization of Tumor Cells in Head and Neck Squamous Cell Carcinoma

**DOI:** 10.3390/biomedicines13020358

**Published:** 2025-02-04

**Authors:** Julia Federspiel, Teresa Bernadette Steinbichler, Samuel Moritz Vorbach, Marie Theres Eling, Wegene Borena, Christof Seifarth, Benedikt Gabriel Hofauer, Jozsef Dudas

**Affiliations:** 1 Department of Otorhinolaryngology and Head and Neck Surgery, Medical University Innsbruck, Austria and University Hospital of Tyrol, 6020 Innsbruck, Austria; teresa.steinbichler@i-med.ac.at (T.B.S.); benedikt-gabriel.hofauer@i-med.ac.at (B.G.H.); jozsef.dudas@i-med.ac.at (J.D.); 2 Department of Radiation-Oncology, Medical University Innsbruck, 6020 Innsbruck, Austria; samuel.vorbach@i-med.ac.at (S.M.V.); marie.eling@i-med.ac.at (M.T.E.); 3 EXTRO-Lab, Tyrolean Cancer Research Institute, Department of Therapeutic Radiology and Oncology, Medical University Innsbruck, 6020 Innsbruck, Austria; 4 Division of Virology, Department of Hygiene, Microbiology, Social Medicine, Medical University Innsbruck, 6020 Innsbruck, Austria; wegene.borena@i-med.ac.at; 5 Institute for Clinical and Functional Anatomy, Medical University Innsbruck, 6020 Innsbruck, Austria; christof.seifarth@i-med.ac.at

**Keywords:** patient-derived cell culture, cancer-associated fibroblasts (CAFs), head and neck squamous cell carcinoma (HNSCC), irradiation, tumor cell relapse

## Abstract

**Background:** The crosstalk between cancer-associated fibroblasts (CAFs) and tumor cells promotes proliferation, tumor relapse, and the acquisition of a partial epithelial-to-mesenchymal (pEMT) phenotype in tumor cells. The aim of this study was to investigate the effects of patient-derived CAFs on tumor cell growth and radioresistance in head and neck squamous cell carcinoma (HNSCC). **Methods:** CAFs were isolated and cultured in a three-dimensional spheroid formation. SCC-25 tumor cells educated by the CAFs (SCC25-E cells) were subjected to irradiation, and the response of the CAF-stimulated tumor cells to radiotherapy was determined using an MTT assay, a clonogenic assay, and Western blotting. Tumor cell morphological changes and growth dynamics were assessed using 3D holotomographic microscopy and a live video microscope. **Results:** Patient-derived CAFs significantly increased the growth rate of SCC-25 cells. CAFs drove fibrosis in the tumor microenvironment (TME), functioned as a physical barrier, temporarily stopped tumor growth, and induced the p38 mitogen-activated protein kinase (MAPK) signaling pathway. Viability after irradiation at 4–8 Gy was significantly higher in SCC25-E cells than in the controls (*p* = 8 × 10^–4^ or lower). Furthermore, irradiation triggered the pEMT profile in HNSCC cells. **Conclusions:** CAFs’ education of tumor cells and the induced p38 phosphorylation had no influence on irradiation sensitivity. SCC25-E cultures demonstrated increased tumor cell growth, viability, and stress-induced phospho-p38 activation.

## 1. Introduction

Similarly to other cancers, HNSCC tumors have high intra- and interindividual variance. Apart from tumor cell populations’ high genetic and epigenetic diversity, tumor progression and resistance to treatment depend on the interactions of HNSCC cells with the surrounding tumor microenvironment (TME) [1]. The TME of HNSCC is a complex system consisting of different stromal cells, including cancer-associated fibroblasts (CAFs), tumor-infiltrating lymphocytes, endothelial cells, adipocytes, and the interstitial extracellular matrix (ECM). CAFs, a specialized and activated group of fibroblasts, constitute the largest component of the tumor stroma in solid tumors such as HNSCC. There exist three different subtypes of CAFs: myofibroblasts (myCAFs), inflammatory CAFs (iCAFs), and antigen-presenting CAFs (apCAFs) [1,2].

CAFs exhibit a different phenotype than normal fibroblasts (NFs); they have higher proliferation levels, increased collagen production, and a distinct secretion of molecules [2,3]. Numerous studies indicate that CAFs play a particularly critical role in tumor growth, metastatic capacity, and therapy resistance by acting as a physical contact barrier to therapeutic agents and immune cell infiltration [4]. Furthermore, CAFs produce chemical tumor mediators altering the TME, including cytokines, chemokines, exosomes, and growth factors, such as transforming growth factor-β (TGF-β), which contribute to tumorigeneses and pEMT in HNSCC [5,6]. In HNSCC, pEMT is not complete; epithelial tumor cells gain mesenchymal marker expression but do not completely switch to a mesenchymal phenotype, as they retain epithelial properties. pEMT was discussed in detail in our previous publication [7]. pEMT cells quickly switch between invasive dissemination and proliferative expansion and vice versa. The pEMT phenotype is responsible for the rapid dissemination of tumor cell groups, as well as for the efficient settlement and expansion of tumor cells during extracapsular spread and metastasis [8,9]. The pro-EMT transcription factor (TF) Slug and pEMT were reported to be associated with radiochemotherapy resistance (RCTR) [6,10,11]. Our experimental data indicate that pEMT in human papilloma virus (HPV)-negative HNSCC tumor cells initially upregulates p38 MAPK signaling and stabilizes Slug protein levels [7]. While epithelial tumor cells undergo pEMT, normal fibroblasts are simultaneously reprogrammed into myCAFs, the largest subset of CAFs within the TME. The main mediator for this reprogramming is TGF-β, of which the tumor cells themselves are a potential source. Furthermore, exosomes produced by tumor cells can trigger the phenotypic alteration from NFs to CAFs [12,13].

To model the relationship between CAFs and HNSCC cells, patient-derived fibroblasts that emerged from a tumor spheroid were cultured, and SCC-25 oral squamous cell carcinoma cells were added to them. SCC-25 cells were originally isolated from the tongue of a 70-year-old male patient and carry a deleterious TP53 mutation [14,15]. According to our previous results [7], SCC-25 cells have the pEMT properties of HPV-negative HNSCC.

The SCC-25 cells overgrew the CAFs and were further cultured, visualized, and prepared for radiation treatment. No traces of CAFs were found in the outgrowth cultures. Nevertheless, substantial changes in the SCC-25 cells were sustained. Tumor-specific growth effects were estimated with and without contact with CAFs. In this study, we hypothesized that CAFs generate fibrosis in the TME to create a physical barrier and trigger the pEMT program via the p38 MAPK signaling pathway. This protein constellation might be triggered by mechanical stress within the tumor cell nest caused by CAFs from the local microenvironment.

## 2. Materials and Methods

### 2.1. Patient Information

A male patient diagnosed with primary oropharynx carcinoma (aged 54 years) consented to CAF isolation and three-dimensional spheroid formation. Available routine immunohistochemistry (IHC) data showed high Slug protein levels and p53 irregularities in the formalin-fixed paraffin-embedded tumor tissue. The tumor tissue was determined to be p16-positive using IHC and HPV-DNA-negative using PCR. These data showed a discrepancy between p16 IHC and HPV-PCR in this patient. The treatment modalities were upfront surgery and post-operative radiotherapy (PORT), and the patient had a complete remission. Relevant clinical data were obtained from the clinical tumor registry from the Department of Otorhinolaryngology, Head and Neck Surgery, Medical University Innsbruck. Permission was obtained from the local ethics committee to collect pretreatment biopsy samples for molecular biological investigation, paraffin embedding, sectioning, and immunohistochemical analysis (Reference Number: UN4428 303/4.14, 26 July 2011). Cell culturing from biopsy samples was approved by the Ethics Committee of the Medical University of Innsbruck (EC number: 1199/2019). Written informed consent was obtained from the patient.

### 2.2. Cell Line

SCC-25 cells (Cat. Nr. ACC 617) were purchased from the German Collection of Microorganisms and Cell Cultures (DSMZ, Braunschweig, Germany) and cultured in DMEM/Ham’s F12 medium, supplemented with 10% FBS, 2 mM L-glutamine, 100 units/mL penicillin, and 100 μg/mL streptomycin (all from Pan-Biotech, Aidenbach, Germany), as well as 1 mM sodium pyruvate and 1x MEM non-essential amino acids (Cat. Nr. P08–32100, Pan-Biotech, Aidenbach, Germany) [5,8].

### 2.3. Spheroids, CAFs, and SCC-25 Cells in CAF Culture

To generate spheroid and CAF cultures, tissue was submerged in DMEM-F12 containing 1% penicillin–streptomycin–amphotericin B (P-S-A) (Cat. Nr. P04-41550, PAN-Biotech, Aidenbach, Germany) and minced. Tissue pieces < 2 mm were pushed three times through 18G needles attached to 2 mL syringes and placed into Sarstedt Biofloat (Sarsted AG & Co. KG, Sarstedt, Germany) 96-well culture plates for spheroid formation. Spheroids were cultured in optimized Epicult Plus medium (Stemcell Technologies, Cat. Nr. 06070; Köln, Germany) for one week. After that, some of them were cryopreserved in Cryostore (Stemcell Technologies CS10, Cat. Nr. 100-1061, Vancouver, Canada) using a slow-cooling procedure (−80 °C at a freezing rate of −1 °C per minute for one day in a CoolCell LX cell freezing container, Corning, Big Flats, NY, USA), followed by storage in liquid nitrogen. The remaining spheroids were suspended by pushing them through 18G needles attached to 2 mL syringes and centrifuged at 4 °C, 290 g for 5 min. The resulting pellet was resuspended and cultured in Fibroblast Growth Medium supplemented with 10% FBS (Cat. Nr. C-23020, Promocell, Heidelberg, Germany) in a 25 cm^2^ culture dish (Sarstedt, Sarstedt, Germany).

For the first week, 10 µM Rho-associated kinase (ROCK) inhibitor Y-27632 (Enzo Life Science, Lörrach, Germany) was added to the culture according to a previously reported method [16]. Fibroblastic cells were detected after 2–3 weeks and were cultured until they reached half-confluency. After a stable culture of carcinoma-associated fibroblasts (CAFs) was obtained, the Fibroblast Growth Medium was replaced by DMEM/F12 (Cat. Nr. P04-41550, PAN-Biotech, Aidenbach, Germany) supplemented with 10% FBS (Cat. Nr. 10270-106), Invitrogen, Paisley, UK). CAFs could be cryopreserved and thawed several times; the culture was reproducible. The whole procedure was repeated, starting from the cryopreserved spheroids, and the CAF culture was also reproduced with the same morphological and culture characteristics.

Five hundred counted (Neubauer chamber, Marienfeld, Lauda-Königshofen, Germany) SCC-25 cells were added to the semiconfluent primary culture of CAFs and cultured in DMEM/F12 supplemented with 10% FBS. The first tumor cell nests were detected after one week and filmed at different growth stages by using a JuliBR04 video microscope (Nanoentek, Seoul, Republic of Korea). The cultured cells’ morphological changes and growth dynamics were assessed using a Juli BR04 live video microscope.

Later, the CAFs did not grow; the CAF/SCC-25 mixed culture became confluent, and passages were needed. After passage 3, CAFs were only sporadically detected. CAFs were completely overgrown by the SCC-25 cells. Passaged SCC-25 cells educated by CAFs (SCC25-E) were used in further radiation therapeutic treatments and Western blotting.

SCC25-E cells were prepared as cell pellets for the HPV-DNA-PCR test. The cell pellets were rinsed with 200 µL of PBS. DNA was isolated using the extraction platform eMAG (Biomerieux Austria, Vienna, Austria). HPV-PCR was conducted using the amplification and hybridization kit HPV-TYPE EXPRESS from AB Analitica (Padova, Italy) according to the manufacturer’s instructions [17].

RNA was isolated and reverse-transcribed from the original tumor sample, SCC-25 cells, CAFs, and tumor cells growing from the CAF/SCC-25 mixed culture, as reported in 2021 [18]. The whole length of the p53 coding sequence was amplified using PCR and sequenced as previously described [18].

### 2.4. Cancer Cell Nest Growth Evaluations Using Live Videos

The growth of the plated SCC-25 cells in the CAF culture was quantified using Image J (ImageJ 1.46r, National Institutes of Health, Bethesda, MA, USA). After finding a few cells containing cancer cell nests, they were photographed with the Juli BR04 video microscope and filmed for 3 days. The frames of the film were taken every 20 min. The area of the growing cancer cell nest was determined by the user in Image J after labeling the tumor cell group in the image. The cancer cell nest areas were identified in all frames for up to 3 days. Time–cancer-cell-nest-area relationships were plotted in Graphpad Prism (10.1.2, Graphpad Software, Boston, MA, USA). The growth curves were regressed using polynomial regression.

### 2.5. Three-Dimensional Holotomographic Microscopy

For 3D holotomographic microscopy, the mixed culture of patient-derived cancer-associated fibroblasts and SCC-25 cells was plated in ibidi dishes with a diameter of 35 mm (ibidi Ltd., Planegg, Germany), as described above. The cells were visualized in different stages of culture. Next, the cells were analyzed for their morphology and interaction under a 3D Cell Explorer-fluo holotomographic microscope (Nanolive SA, Tolochenaz, Switzerland) with an air objective at 60× magnification. Typical histomorphologic features of CAFs and cancer cells were assessed. Images were collected for further computational analysis using STEVE software Version 1. 6. 3496 (Nanolive SA, Tolochenaz, Switzerland).

### 2.6. Experimental Radiation Therapy

SCC25-E cells growing out from the CAF/SCC-25 mixed culture and the original SCC-25 cell line were plated at 10^4^ cells per mL in 25 cm^2^ culture dishes, 5 mL of cell suspension per dish, in DMEM/F12 supplemented with 10% FBS for 3 days. Before irradiation, the medium was changed. We irradiated 2 dishes of SCC-25 and mixed-culture-based cells (SCC25-E) with 0 Gy (control), 2 Gy, 4 Gy, 6 Gy, 8 Gy, and 10 Gy in one fraction with a dose rate of 2.7 Gy per minute. A 6 MV photon beam from a Varian Clinac 2100 linear accelerator (Varian Medical Systems, Inc., Palo Alto, CA, USA) was used. After radiation treatment, the cells were removed from the culture dishes by performing two PBS washes and trypsin incubation (Cat. Nr. P10-023100, PAN-Biotech, Aidenbach, Germany) and plated for the MTT assay to determine metabolic activity [7,11] and clonogenic survival (10^3^ cells in 25 cm^2^ culture dishes, two dishes per treatment [11]), as described previously. For Western blotting, 3 × 10^5^ cells per mL were plated in 25 cm^2^ culture dishes. All cultures were grown in DMEM/F12 supplemented with 10% FBS. The MTT assay was completed 4 days after plating, Western blotting was performed 1 week after plating, and clonogenic survival was evaluated 2 weeks after plating using gentian violet, as previously described [11].

The quantitative results of radiation were visualized by plotting dose–effect curves. As a measure of potential radioprotection resulting from the co-culture of CAFs/SCC-25, dose-modifying factors (DMFs) were calculated from the results of the clonogenic assays of CAF-educated SSC-25 cells, as described by Rosenberg [19]. DMFs were calculated as the dose required to achieve 10% iso-survival in CAF/SCC-25 co-cultured cells divided by the dose required to achieve the same survival rate in the SCC-25 mono-culture control cells (DMF10) [11]. To additionally determine a possible difference in the dose-modifying effect between the co-culture and mono-culture in the low-, mid-, or high-dose range, the mean DMF90 and DMF50 (90% and 50% iso-survival) were calculated and compared to those of DMF10.

### 2.7. Flow Cytometric Assessment of Passaged Cultured Cells

The passaged SCC25-E cells were collected from culture dishes by performing trypsinization (Cat. Nr. P10-023100, PAN-Biotech, Aidenbach, Germany) and characterized via flow cytometry using the Beckman Coulter PerFix NC (Beckman Coulter, Brea, CA, USA) kit following the instructions of the manufacturer. Directly conjugated antibodies were used: Pan-Cytokeratin-FITC (Cat. Nr. IM2356U, 10 µL per sample; Beckman Coulter, Brea, CA, USA) and p16-FITC (Cat. Nr. 556560, BD, Pharmingen, San Diego, CA, USA). Flow cytometry data were analyzed using CytExpert 1.2 (Beckman Coulter, Brea, CA, USA) as reported previously [7].

### 2.8. Western Blot

After the treatments were completed, the cells were scraped into RIPA buffer [7,8] and used for protein isolation and Western blotting. Rabbit monoclonal primary antibodies against p-p38 MAPK (Cat. Nr. 4511S, 1:1000) and vimentin (SP-20, 1:100) were purchased from Cell Signaling Technology (Danvers, MA, USA) and Spring Biotech (Espoo, Finland).

For signal detection, highly specific isotype-matching secondary antibodies (anti-rabbit) conjugated with either peroxidase (Cat. Nr. 31460, Invitrogen, Darmstadt, Germany) or a near-infrared (NIR) fluorescence marker (Cat. Nr. 926-32213, Li-cor Biosciences, Lincoln, NE, USA) (1:1000 and 1:10,000 dilutions) were used in conditions recommended by the manufacturers. GAPDH protein levels were used as loading controls and were detected with an anti-GAPDH antibody (Cat. Nr. ab8245, dilution 1:5000) purchased from Abcam (Cambridge, UK). For GAPDH detection, fluorescence-conjugate-labeled anti-mouse IgG secondary antibodies, available from Li-cor, were used. The Azure C500 Western blot imaging biosystem (Azure Biotech, Houston, TX, USA) was used to visualize specific NIR fluorescence and chemiluminescence signals. Western blot bands’ optical density measurements were carried out as previously described [18].

### 2.9. Immunohistochemistry and Image Acquisition

IHC for phosphorylated p38 MAPK was performed using the rabbit monoclonal primary antibody against p-p38 MAPK (Cat. Nr. 4511S; 1:400), purchased from Cell Signaling Technology (Danvers, MA, USA). The staining reaction was developed using a universal secondary antibody (Ventana, Tucson, AZ, USA) and the DAB Map kit of Ventana, as described previously [18,20,21]. The IHC labeling levels were acquired in TissueFax systems (TissueGnostics, Vienna, Austria, Software Version 7.1) in brightfield using a Pixelink camera, model PL-D674CU-CYL-07451 (PIXELINK; Rochester; NY, USA) [5,8].

### 2.10. Data Analysis

All experiments were repeated three times. The data sets were tested for normal distributions using the D’Agostino and Pearson omnibus normality test. Comparisons of two data sets were carried out using Student’s t-test for normally distributed data or with the Wilcoxon matched-pairs signed-rank test for non-parametric data. Comparisons of more than two data sets were conducted by performing a parametric ANOVA or non-parametric Kruskal–Wallis test. Statistical analysis was performed with SPSS Version 29 (IBM, Chicago, IL, USA) and Graphpad Prism Version 10 (Graphpad Software Inc., San Diego, CA, USA).

## 3. Results

### 3.1. Characterization of Patient-Derived Cell Culture

As shown in Supplementary Document S1, the original patient’s oropharynx squamous cell carcinoma contained an I232F loss-of-function p53 mutation, which was not present in the cultured cells. This meant that the tumor cells of the patient were not cultured, only the CAFs, which did not contain this p53 mutation. In mixed culture, the SCC-25-specific p53 codon 209AGA: A and G double deletions were detected by Sanger sequencing, but the patient-specific loss-of-function mutation I232F was not present. This meant that the cultured CAFs from the patient did not come from the p53-mutated tumor or SCC-25 cells, whose origin was evidenced by p53 Sanger sequencing (Supplementary Document S1). The CAF/SCC-25 culture did not contain the p16 protein (not detected in the Western blot) or HPV-specific DNA (analyzed by the Division of Virology; Medical University Innsbruck). Since the original tumor is p16-positive (and HPV-PCR-negative), this was additional evidence that we did not culture the original tumor cells from the patient, only the CAFs, which were p16-negative. The passaged, CAF-educated SCC-25 cells were also subjected to FACS analysis and showed Cytokeratin-positive and p16INK4-negative reactions (Supplementary Document S2, Figure S1).

### 3.2. Patient-Derived CAF Behavior and the Potential to Influence Tumor Cell Growth in HNSCC

**Figure 1:**
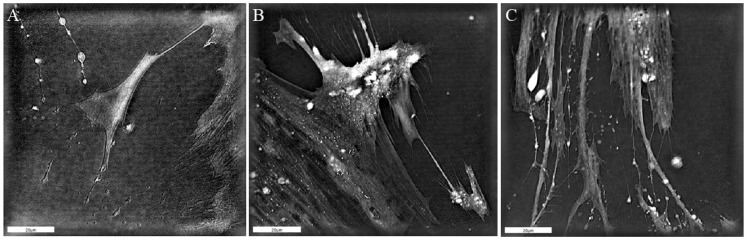
Holotomographic images of patient-derived CAF morphology in vitro. (**A**) An example of adherent–fibrotic, stellate-shaped morphology. (**B**) Intermediate CAFs with elongated and star-like cell morphology. (**C**) Invasive–migratory spindle-shaped morphology. Brighter regions correspond to areas of higher dry mass density. A 90 × 90 µm field of view is shown.

CAFs in the surrounding stroma in the TME contribute to tumor progression by promoting tumor growth [3,22]. CAFs were isolated and cultured from a patient with confirmed oropharyngeal squamous cell carcinoma. The primary culture was grown for 6 days in the form of a 3D spheroid and then cultured on plastic in 2D, in which only CAFs, but not tumor cells, grew. In the patient-derived 2D culture, two different types of CAFs could be identified: adherent–fibrotic and invasive–migratory CAFs with a higher proliferative potential. In this phase, the CAFs displayed morphological changes from a stellate-shaped (Figure 1A) to a protracted spindle-shaped cell morphology (Figure 1B,C) with central cell nuclei. To establish the CAF tumor cell model, 500 SCC-25 tumor cells were transferred into the CAF culture.

**Figure 2:**
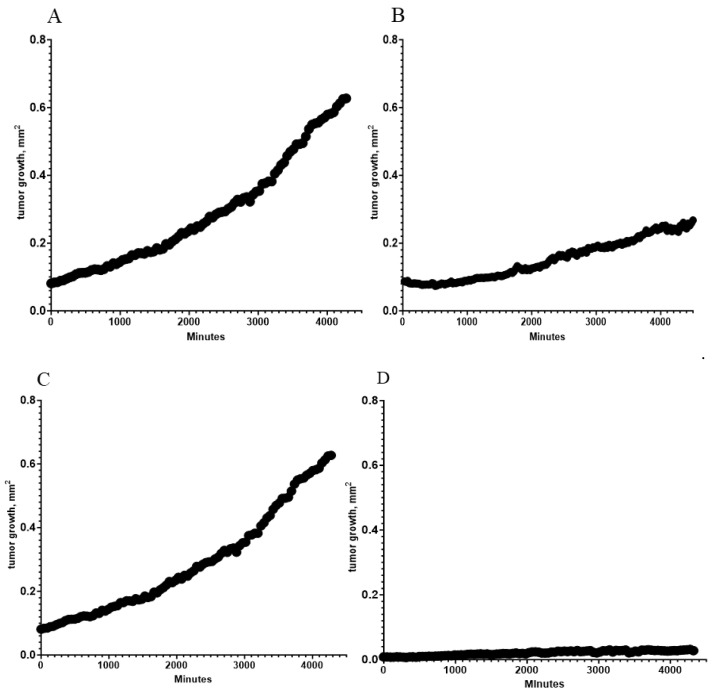
The tumor cell growth of SCC-25 cells in the mixed culture. (**A**) SCC25 cells in the presence of patient-derived CAFs and (**B**) without CAFs. (**C**) Differences in the tumor cell growth of the mixed culture without and (**D**) with direct physical interaction with the CAFs. The cell growth in all graphs is described by second-order polynomial fitted curves with (**A**,**C**) R^2^ = 0.996, (**B**) R^2^ = 0.991, (**D**) R^2^ = 0.935.

The introduction of the tumor cells into the CAF culture resulted in a rapidly proliferating tumor cell colony without direct physical contact with the tumor cell nest. Compared to the control (SCC-25 cells), the tumor cell growth rate of the mixed culture was doubled (SCC-25 cells in normal plastic culture: 0.12 mm^2^/day; SCC-25 cells supported by CAFs: 0.2 mm^2^/day). This was based on the regression and resulting area-growth/time equations of the growth curves presented in Figure 2A,B. For all curves, the best curve fit was the second-order polynomial (quadratic).

In the next step, the direct and indirect physical growth effects of CAFs on the tumor cell nest were investigated (Figure 2C,D). During tumor progression, the CAFs built up a physical barrier, forming a capsule that enclosed the tumor cell nest, which temporally stopped tumor cell growth (Figure 2D). The tumor cell growth rate with the CAF barrier was reduced to 0.016 mm^2^/day, which was 8.09% of the tumor cell growth of cell colony without direct physical contact with CAFs.

**Figure 3:**
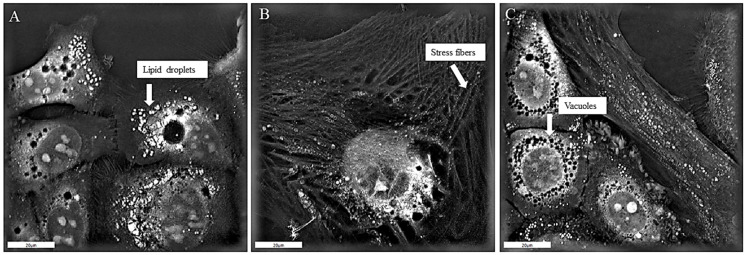
Holotomographic imaging of tumor cells with direct physical contact with CAFs. (**A**) Tumor cells containing lipid droplets (bright white dots) and vacuoles of different sizes (black circles). Fine adhesions can be seen attaching cells to the plate surface. (**B**) An example of a tumor cell with increased surface area, the appearance of stress fibers (straight-line structures), and further membrane disruption. (**C**) Tumor cells containing small vacuoles, seen as black circles (left). The disruption of tumor cell plasma membranes is also visible as brighter areas around the tumor cell perimeter. Brighter regions correspond to areas of higher dry mass density. A 90 × 90 µm field of view is shown.

Holotomographic microscopy (HT) allowed a causal link to be established between direct physical contact with CAFs and the gain of the invasive phenotype in tumor cells. The results showed that when the tumor cells were exposed to the CAFs, the organization of the tumor cell nuclei tended toward polyploidy, cytoplasmic vacuoles, and lipid droplets were observed, as demonstrated in Figure 3A. The presence of straight-lined stress fibers around the tumor cells and cell morphological changes due to plasma membrane disruption were further characteristics of the tumor cells embedded in the CAF population (Figure 3B). Moreover, there were clear signs of membrane movement and mechanisms of cell membrane ruffling; numerous thin membrane protrusions where cells were attached to the surface and to each other; and large, pale, circular structures around the cell edges (Figure 3C). Nonapoptotic membrane blebbing plays a central role in cancer cell migration and invasion [23,24]. Zoomed-in subfigures of the highlighted structures are presented in Supplementary Document S2, Figure S2.

### 3.3. Clonogenic Survival and Cell Viability After Radiation of CAF-Educated SCC-25 Cells

**Figure 4:**
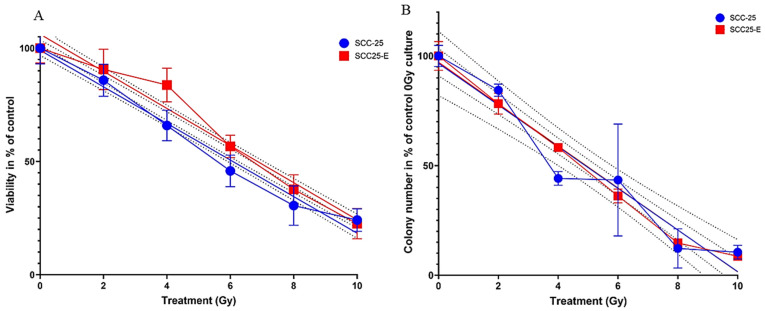
Irradiation of SCC-25 and SCC25-E cells with 0–10 Gy. (**A**) Relative values of metabolic cell activity, determined by MTT assay, and (**B**) clonogenic survival, both related/normalized to unirradiated control cultures. SCC25-E cells had significantly higher metabolic capacity after irradiation with 4–8 Gy, *p* = 8 × 10^–4^ or lower, with Mann–Whitney-test, *n* = 24 in all cases. Error bars: standard error of measurement.

SCC-25 and SCC25-E cells were treated with single doses of 0–10 Gy, followed by replating for the MTT assay and clonogenic survival analysis. The MTT assay revealed significantly increased cell survival at 4–8 Gy for SCC25-E cells (*p*-values with Mann–Whitney test for 4 and 6 Gy: *p* < 10^−4^; for 8 Gy: *p* = 8 × 10^−4^; Figure 4A); the clonogenic survival analysis did not detect clear differences between SCC-25 and SCC-25E cultures (Figure 4B). The radiosensitivity of SCC25-E cultures versus reference SCC-25 mono-cultures was expressed by calculating DMF10, DMF50, and DMF90. A negligible dose-modifying factor of 1.01 was determined to achieve 10% clonogenic iso-survival in SCC25-E cultures and SCC-25 mono-cultures (DMF10). Analogous values were determined for 50% and 90% clonogenic iso-survival (DMF50 = 1.01; DMF90 = 1.02). These results indicate that CAF education does not have a significant protective effect on the clonogenic survival of irradiated SCC-25 cells.

### 3.4. Radiation Sustains Pospho-p38 Signaling and Promotes pEMT

**Figure 5:**
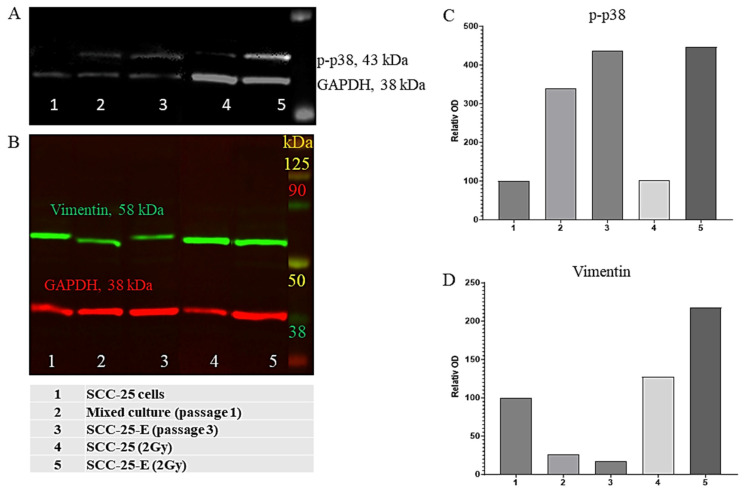
Western blot analysis of p-p38 and vimentin in SCC-25 and SCC25-E cells with and without irradiation. The original Western blot image of (**A**) p-p38 and (**B**) vimentin compared to the loading control GAPDH in control SCC-25 cells (1), in the mixed culture at passages 1 (2) and 3 (3), in irradiated SCC-25 cells (4), and in irradiated SCC25-E cells (p3) (5). Densitometry of (**C**) p-p38 and (**D**) vimentin normalized to the GAPDH loading control. The normalized optical density of the control SCC-25 cells was considered 100%, and the columns show the relative normalized optical density.

As reported in 2022 [8], the maintenance of phospho-p38 (p-p38) is essential for the therapy-resistant pEMT phenotype in HNSCC. Cisplatin treatment of SCC-25 cells induced p38–MAPK phosphorylation in surviving tumor cells [7]. To investigate p-p38 signaling in response to CAFs and radiation, SCC-25 cells (control), tumor cells of the mixed culture (passage 1), and SCC25-E cells (passage 3) with and without radiation treatment were prepared for Western blot detection. The interaction with CAFs clearly resulted in a 3–4-fold increase in p-p38 levels in SCC25-E compared to the control (Figure 5A,C). Moreover, p-p38 levels remained high after radiation (2 Gy) in the SCC25-E cells (Figure 5A,C). The Western blot analysis of vimentin protein levels in the mixed culture (passage 1) and SCC25-E cells showed a tendency toward downregulation, which supports the epithelial character of the CAF-influenced tumor cells compared to the control (SCC-25). In contrast, both SCC-25 and SCC25-E tumor cells (2 Gy) gained a pEMT profile after irradiation, resulting in high and stable vimentin protein levels (Figure 5B,D). Interestingly, the low level of vimentin in SCC25-E cells increased 8-fold after irradiation (Figure 5D).

### 3.5. Detection of p38 MAPK Phosphorylation in the HNSCC Patient Tumor Tissue

**Figure 6:**
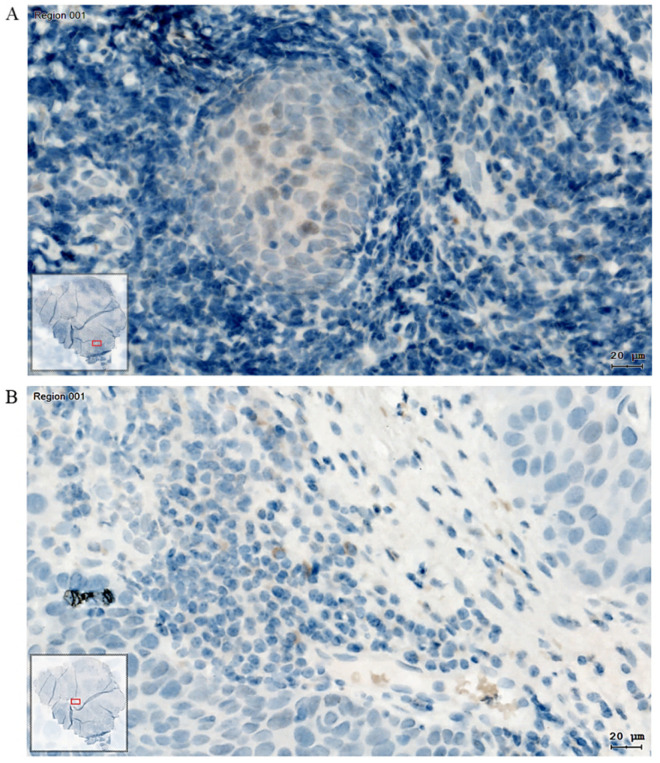
(**A**) Identification of the brown positive staining reaction of p-p38 in the nuclei of tumor cells in the original HNSCC cancer cell nest. (**B**) Cytoplasmic p-p38 antigen detection of single cells in the stromal compartment of the sectioned HNSCC tissue. Brown reactions without connections to cell nuclei are artifacts. The bottom left box of each image shows an overview of the whole scanned tissue section. The red box in each image shows the location of the presented area in the original tissue section.

The paraffin-embedded tumor tissue sample of the patient, who was the source of the CAFs, was immunostained for p-p38. In this HNSCC tissue specimen, some tumor cell nuclei and some stromal cell cytoplasm displayed the activated, phosphorylated form of p38 (Figure 6A,B). Sporadic p-p38-positive cells were present in the whole tissue. In addition, in some tumor cell nests, which were embedded in a cell-rich stroma (Figure 6A), the positive nuclear staining reaction was more accumulative than in the rest of the tissue. Additionally, p-p38 was located in the cytoplasm of stromal cells in the TME (Figure 6B). Moreover, single migratory tumor cells and cells with elongated cell nuclei represent a p-p38-positive staining reaction (Figure 6A).

## 4. Discussion

CAFs are a major component of the tumor microenvironment and promote tumorigenesis. CAFs are present in approximately 90% of epithelial tumors and have an intimate association with tumor initiation and progression [25]. CAFs are derived either from normal fibroblasts, pericytes, smooth muscle cells, endothelial cells, or mesenchymal stem cells or from epithelial cancer cells via pEMT [26]. CAFs’ heterogeneity and plasticity generate a challenge in determining their precise cellular origin [2]. CAF subtypes show a dynamic nature, representing a hybrid cellular state rather than a final end-point of differentiation [27]. As a substantial cell population in the TME, CAFs can be simply defined as the mesenchymal fibroblastic cell type surrounding the tumor tissue [26].

Several cell surface, intracellular, and extracellular proteins were used to identify CAFs and separate them from normal fibroblasts [1,28]. In our pre-experimental setting, CAFs were identified by using flow cytometry to detect the three common CAF markers: alpha smooth muscle actin (αSMA), fibroblast activation protein alpha (FAP), and Podoplanin. The large spindle-shaped cell morphology and the lack of genetic mutations found within tumor cells are further required to ensure the identification of CAFs [27]. Genetic anomalies that are present in the tumor cells and the CAFs are rarely similar, suggesting that only a small population of tumor cells and CAFs share a common origin. The isolated patient-derived CAFs lacked both the I232F loss-of-function p53 mutation and p16 overexpression verified in the HNSCC patient profile.

Alterations in the tumor suppressor protein p53 might critically influence the regulation of CAFs. According to Liu et al., irregular p53 triggers the pro-tumorigenic activation of CAFs via the Stat 3 signaling pathway, resulting in the enhanced expression of CAF-specific markers and the production of ECM [29].

The above results are in line with recent studies [30,31], suggesting that mutations in the p53 protein-coding region in pancreatic cancer cells promote an antiapoptotic phenotype in CAFs, capable of establishing a tumor-supportive and pro-metastatic environment. Moreover, irregular p53 functionality in CAFs mediated cellular senescence and enhanced the expression of senescence-related genes in the CAF population [32,33]. CAFs are described as particularly radioresistant, as they survive radiation but enter cellular senescence [26,34]. This therapy-induced senescence can affect the malignant potential of CAFs in promoting tumorigenesis [3,35]. In our experimental setting, CAFs isolated from a patient with oropharyngeal cancer supported cancer cell growth, with tumor cell growth doubling (SCC-25 cells in normal plastic culture: 0.12 mm^2^/day; SCC-25 cells supported by CAFs: 0.2 mm^2^/day). CAFs in direct contact with tumor cells built a physical barrier, forming a capsule that enclosed the tumor cell nest, which temporally stopped tumor cell growth (Figure 2D). The tumor cell growth rate was reduced to 0.016 mm^2^/day, which was 8.09% of the tumor cell growth without the CAF barrier.

Additionally, CAFs did not influence the sensitivity of SCC-25 cells to irradiation or increase their radioresistance. There are several publications discussing the effects of CAFs on tumor cells under radiotherapy conditions, where the main observations are the robust radioresistant survival of CAFs and their increased TGF-β production after irradiation, which might result in increased tumor cell resistance as well [26]. In our culture system, the CAFs were only sporadically present in passaged mixed cultures and disappeared in passage 3. The increased seeding capacity of SCC25-E cells was detectable in control (0 Gy) treatment conditions (313 vs. 215 colonies) and remained increased in radiated conditions (Figure 4A). In order to calculate the radiation effects, the treated cells were compared to 0 Gy controls, with the difference expressed in percent. This comparison did not show increased radioresistance, which means that the CAF co-culture did not change the tumor cells’ reactivity to radiation. As reported in a previous study, CAFs and their interaction behavior may have no effect on radioresistance [36].

Interestingly, CAF interaction induced growth stress in SCC-25 cells and the irradiation of SCC25-E cells sustained p38-MAPK-signaling (Figure 5A,C). In addition, irradiation-induced pEMT in SCC-25 and SCC25-E cells. The relationship between irradiation-induced pEMT and increased p38-MAPK-signaling in HNSCC has been discussed [37]. In terms of radiosensitivity, the role of p38-MAPK-signaling is still unclear [37,38].

In paraffin sections of the original patient HNSCC tissue, we detected encapsulated cancer cell nests with increased p-p38 signals. In this context, using the patient-isolated CAFs in our model successfully reproduced one of the CAF effects observed in the patient. Since we were unable to culture the patient’s original tumor cells, we used SCC-25 cells, a well-established model for HNSCC.

The original primary tumor was excised surgically, followed by PORT. PORT is used to treat remaining low-quantity tumor cells, which are responsible for recurrence. Tumor recurrence was not detected several months after treatment in the patient. This might indicate that, although the p-p38 signal was increased in some cancer cell nest clusters, it did not lead to increased radioresistance, or the propagation of the resistant cells might take longer than the available 9 months. Experimentally, the seeding of passaged SCC25-E cells was improved in irradiated settings, as mentioned above. Although CAFs induced the doubling of SCC-25 tumor cell growth with increased proliferation, the cells did not become more sensitive to irradiation [13].

CAFs sustained phospho-p38 signaling in both epithelial and pEMT-SCC-25 cells. This background might maintain, but not increase, the radioresistance of SCC-25 tumor cells (approximately 50% of the SCC-25 cells survive 4 Gy) through a direct mechanism, such as sustaining p-p38 signaling and promoting pEMT. This point was also discussed before [37].

In our culture system, patient-derived CAFs supported the colonization of HNSCC tumor cells. In conclusion, our findings demonstrate that CAFs promote the resettlement of tumor cells via the p-p38 pathway, suggesting that CAFs contribute to tumor relapse in HNSCC.

## Data Availability

All data supporting the reported results are contained within the article or Supplementary Materials Documents S1 and S2. The original data presented in the study are openly available in Zenodo at doi:10.5281/zenodo.14689809.

## Supplementary Materials

The following supporting information can be downloaded at https://www.mdpi.com/article/10.3390/biomedicines13020358/s1: Document S1: Sequencing data; Document S2: FACS analysis of CAF-educated SCC-25 cells.

## Abbreviations

The following abbreviations are used in this manuscript:

CAFs	Cancer-associated fibroblasts
DMF	Dose-modifying factors
HT	Holotomographic microscopy
ECM	Extracellular matrix
NFs	Normal fibroblasts
pEMT	Partial epithelial–mesenchymal transition
PORT	Post-operative radiotherapy
p38-MAPK	p38 mitogen-activated protein kinase
TME	Tumor microenvironment
