# Supplementary material for: Patient-Derived Cancer-Associated Fibroblasts Support the Colonization of Tumor Cells in Head and Neck Squamous Cell Carcinoma
Source: Biomedicines. 2025 Feb 4;13(2):358. doi: 10.3390/biomedicines13020358 (PMC11852712; doi:10.3390/biomedicines13020358)
Supplement: Supplementary file 1 [file biomedicines-13-00358-s001.zip › Supplementary Document S1.pdf]

Supplementary Document S1: Sequencing data

1. **P53 sequence alignment of SCC-25 cells with seq reference: 1126112 p53 standard**

Query: SCC-25 sample; Subject: reference standard.

```
Query  1     CATTTTCAGACCTATGGAAACTACTTCCTGAAAACAACGTTCTGTCCCCCTTGCCGTCCC
60
             ||||||||||||||||||||||||||||||||||||||||||||||||||||||||||||
Sbjct  252   CATTTTCAGACCTATGGAAACTACTTCCTGAAAACAACGTTCTGTCCCCCTTGCCGTCCC
311

Query  61    AAGCAATGGATGATTTGATGCTGTCCCCGGACGATATTGAACAATGGTTCACTGAAGACC
120
             ||||||||||||||||||||||||||||||||||||||||||||||||||||||||||||
Sbjct  312   AAGCAATGGATGATTTGATGCTGTCCCCGGACGATATTGAACAATGGTTCACTGAAGACC
371

Query  121   CAGGTCCAGATGAAGCTCCCAGAATGCCAGAGGCTGCTCCCCGCGTGGCCCCTGCACCAG
180
             |||||||||||||||||||||||||||||||||||||||||| |||||||||||||||||
Sbjct  372   CAGGTCCAGATGAAGCTCCCAGAATGCCAGAGGCTGCTCCCCCCGTGGCCCCTGCACCAG
431

Query  181   CAGCTCCTACACCGGCGGCCCCTGCACCAGCCCCCTCCTGGCCCCTGTCATCTTCTGTCC
240
             ||||||||||||||||||||||||||||||||||||||||||||||||||||||||||||
Sbjct  432   CAGCTCCTACACCGGCGGCCCCTGCACCAGCCCCCTCCTGGCCCCTGTCATCTTCTGTCC
491

Query  241   CTTCCCAGAAAACCTACCAGGGCAGCTACGGTTTCCGTCTGGGCTTCTTGCATTCTGGGA
300
             ||||||||||||||||||||||||||||||||||||||||||||||||||||||||||||
Sbjct  492   CTTCCCAGAAAACCTACCAGGGCAGCTACGGTTTCCGTCTGGGCTTCTTGCATTCTGGGA
551

Query  301   CAGCCAAGTCTGTGACTTGCACGTACTCCCCTGCCCTCAACAAGATGTTTTGCCAACTGG
360
             ||||||||||||||||||||||||||||||||||||||||||||||||||||||||||||
Sbjct  552   CAGCCAAGTCTGTGACTTGCACGTACTCCCCTGCCCTCAACAAGATGTTTTGCCAACTGG
611

Query  361   CCAAGACCTGCCCTGTGCAGCTGTGGGTTGATTCCACACCCCCGCCCGGCACCCGCGTCC
420
             ||||||||||||||||||||||||||||||||||||||||||||||||||||||||||||
Sbjct  612   CCAAGACCTGCCCTGTGCAGCTGTGGGTTGATTCCACACCCCCGCCCGGCACCCGCGTCC
671

Query  421   GCGCCATGGCCATCTACAAGCAGTCACAGCACATGACGGAGGTTGTGAGGCGCTGCCCCC
480
             ||||||||||||||||||||||||||||||||||||||||||||||||||||||||||||
Sbjct  672   GCGCCATGGCCATCTACAAGCAGTCACAGCACATGACGGAGGTTGTGAGGCGCTGCCCCC
731

Query  481   ACCATGAGCGCTGCTCAGATAGCGATGGTCTGGCCCCTCCTCAGCATCTTATCCGAGTGG
540
             ||||||||||||||||||||||||||||||||||||||||||||||||||||||||||||
Sbjct  732   ACCATGAGCGCTGCTCAGATAGCGATGGTCTGGCCCCTCCTCAGCATCTTATCCGAGTGG
791
```

```
Query  541   AAGGAAATTTGCGTGTGGAGTATTTGGATGAC--AAACACTTTTCGACATAGTGTGGTGG  598
             ||||||||||||||||||||||||||||||||  ||||||||||||||||||||||||||
Sbjct  792   AAGGAAATTTGCGTGTGGAGTATTTGGATGACAGAAACACTTTTCGACATAGTGTGGTGG  851

Query  599   TGCCCTATGAGCCGCCTGAGGTTGGCTCTGACTGTACCACCATCCACTACAACTACATGT  658
             ||||||||||||||||||||||||||||||||||||||||||||||||||||||||||||
Sbjct  852   TGCCCTATGAGCCGCCTGAGGTTGGCTCTGACTGTACCACCATCCACTACAACTACATGT  911

Query  659   GTAACAGTTCCTGCATGGGCGGCATGAACCGGAGGCCCATCCTCACCATCATCACACTGG  718
             ||||||||||||||||||||||||||||||||||||||||||||||||||||||||||||
Sbjct  912   GTAACAGTTCCTGCATGGGCGGCATGAACCGGAGGCCCATCCTCACCATCATCACACTGG  971

Query  719   AAGACTCCAGTGGTAATCTACTGGGACGGAACAGCTTTGAGGTGCGTGTTTGTGCCTGTC  778
             ||||||||||||||||||||||||||||||||||||||||||||||||||||||||||||
Sbjct  972   AAGACTCCAGTGGTAATCTACTGGGACGGAACAGCTTTGAGGTGCGTGTTTGTGCCTGTC  1031

Query  779   CTGGGAGAGACCGGCGCACAGAGGAAGAGAATCTCCGCAAGAAAGGGGAGCCTCACCACG  838
             ||||||||||||||||||||||||||||||||||||||||||||||||||||||||||||
Sbjct  1032  CTGGGAGAGACCGGCGCACAGAGGAAGAGAATCTCCGCAAGAAAGGGGAGCCTCACCACG  1091

Query  839   AGCTGCCCCCAGGGAGCACTAAGCGAGCACTGCCCAACAACAYCAGCTCCTCTCCCCAGC  898
             |||||||||||||||||||||||||||||||||||||||||| |||||||||||||||||
Sbjct  1092  AGCTGCCCCCAGGGAGCACTAAGCGAGCACTGCCCAACAACACCAGCTCCTCTCCCCAGC  1151

Query  899   CAAAGAAGAAACCACTGGATGGAGAATATTTCACCCTTCAGATCCGTGGGCGTGAGCGCT  958
             ||||||||||||||||||||||||||||||||||||||||||||||||||||||||||||
Sbjct  1152  CAAAGAAGAAACCACTGGATGGAGAATATTTCACCCTTCAGATCCGTGGGCGTGAGCGCT  1211

Query  959   TCGAGATGTTCCGAGAGCTGAATGAAGCCTTGCAACTCAAGGATGCCCAAGCTGGGAAAG  1018
             ||||||||||||||||||||||||| |||||| |||||||||||||||| |||||||| |
Sbjct  1212  TCGAGATGTTCCGAGAGCTGAATGAGGCCTTGGAACTCAAGGATGCCCAGGCTGGGAAGG  1271

Query  1019  AGCMAGGGGGGAGCAGG  1035
             ||| |||||||||||||
Sbjct  1272  AGCCAGGGGGGAGCAGG  1288
```

Sequence compare on Ensembl.org

http://www.ensembl.org/Homo_sapiens/Transcript/Sequence_cDNA?db=core;g=ENSG00000141510;r=17:7675229-7687427;t=ENST00000269305

Codon 72: CCC changes to CGC: Prolin to Arginine problably unimportant, influences a proline rich region.

Codon 209 AGA : A & G deleted. DELETION causes frameshift and truncated protein occurs DNA binding domain involved. Arginine deleted. End of the protein multiple point mutations.

Protein transcript analysis in SCC-25 cells.

MEEPQSDPSVEPPLSQETFSDLWKLLPENNVLSPLPSQAMDDLMLSPDDIEQWFTEDPGP

DEAPRMPEAAPRVAPAPAAPTPAAPAPAPSWPLSSSVPSQKTYQGSYGFRLGFLHSGTAK

SVTCTYSPALNKMFCQLAKTCPVQLWVDSTPPPGTRVRAMAIYKQSQHMTEVVRRCPHHE

RCSDSDGLAPPQHLIRVEGNLRVEYLDDKHFST - STOP

SCC-25 cells produce this truncated p53 protein, which is absolutely not functional.

2. P53 sequence alignment of original tumor sample from patient oropharynx SSC with seq reference: 1126112 p53 standard

```
Query  1    GGCT-CGGGGA-MCTTTGCGTTCGGGCTGGGAGCGTGCTTTCCACGACGGTGACACGCTT  58
            |||| ||||||  |||||||||||||||||||||||||||||||||||||||||||||||
Sbjct  44   GGCTCCGGGGACACTTTGCGTTCGGGCTGGGAGCGTGCTTTCCACGACGGTGACACGCTT  103

Query  59   CCCTGGATTGGCAGCCAGACTGCCTTCCGGGTCACTGCCATGGAGGAGCCGCAGTCAGAT  118
            ||||||||||||||||||||||||||||||||||||||||||||||||||||||||||||
Sbjct  104  CCCTGGATTGGCAGCCAGACTGCCTTCCGGGTCACTGCCATGGAGGAGCCGCAGTCAGAT  163

Query  119  CCTAGCGTCGAGCCCCCTCTGAGTCAGGAAACATTTTCAGACCTATGGAAACTACTTCCT  178
            ||||||||||||||||||||||||||||||||||||||||||||||||||||||||||||
Sbjct  164  CCTAGCGTCGAGCCCCCTCTGAGTCAGGAAACATTTTCAGACCTATGGAAACTACTTCCT  223

Query  179  GAAAACAACGTTCTGTCCCCCTTGCCGTCCCAAGCAATGGATGATTTGATGCTGTCCCCG  238
            ||||||||||||||||||||||||||||||||||||||||||||||||||||||||||||
Sbjct  224  GAAAACAACGTTCTGTCCCCCTTGCCGTCCCAAGCAATGGATGATTTGATGCTGTCCCCG  283

Query  239  GACGATATTGAACAATGGTTCACTGAAGACCCAGGTCCAGATGAAGCTCCCAGAATGCCA  298
            ||||||||||||||||||||||||||||||||||||||||||||||||||||||||||||
Sbjct  284  GACGATATTGAACAATGGTTCACTGAAGACCCAGGTCCAGATGAAGCTCCCAGAATGCCA  343

Query  299  GAGGCTGCTCCCCGCGTGGCCCCTGCACCAGCAGCTCCTACACCGGCGGCCCCTGCACCA  358
            ||||||||||||| ||||||||||||||||||||||||||||||||||||||||||||||
Sbjct  344  GAGGCTGCTCCCCCCGTGGCCCCTGCACCAGCAGCTCCTACACCGGCGGCCCCTGCACCA  403
```

```
Query  359   GCCCCCTCCTGGCCCCTGTCATCTTCTGTCCCTTCCCAGAAAACCTACCAGGGCAGCTAC  418
             ||||||||||||||||||||||||||||||||||||||||||||||||||||||||||||
Sbjct  404   GCCCCCTCCTGGCCCCTGTCATCTTCTGTCCCTTCCCAGAAAACCTACCAGGGCAGCTAC  463

Query  419   GGTTTCCGTCTGGGCTTCTTGCATTCTGGGACAGCCAAGTCTGTGACTTGCACGTACTCC  478
             ||||||||||||||||||||||||||||||||||||||||||||||||||||||||||||
Sbjct  464   GGTTTCCGTCTGGGCTTCTTGCATTCTGGGACAGCCAAGTCTGTGACTTGCACGTACTCC  523

Query  479   CCTGCCCTCAACAAGATGTTTTGCCAACTGGCCAAGACCTGCCCTGTGCAGCTGTGGGTT  538
             ||||||||||||||||||||||||||||||||||||||||||||||||||||||||||||
Sbjct  524   CCTGCCCTCAACAAGATGTTTTGCCAACTGGCCAAGACCTGCCCTGTGCAGCTGTGGGTT  583

Query  539   GATTCCACACCCCCGCCCGGCACCCGCGTCCGCGCCATGGCCATCTACAAGCAGTCACAG  598
             ||||||||||||||||||||||||||||||||||||||||||||||||||||||||||||
Sbjct  584   GATTCCACACCCCCGCCCGGCACCCGCGTCCGCGCCATGGCCATCTACAAGCAGTCACAG  643

Query  599   CACATGACGGAGGTTGTGAGGCGCTGCCCCCACCATGAGCGCTGCTCAGATAGCGATGGT  658
             ||||||||||||||||||||||||||||||||||||||||||||||||||||||||||||
Sbjct  644   CACATGACGGAGGTTGTGAGGCGCTGCCCCCACCATGAGCGCTGCTCAGATAGCGATGGT  703

Query  659   CTGGCCCCTCCTCAGCATCTTATCCGAGTGGAAGGAAATTTGCGTGTGGAGTATTTGGAT  718
             ||||||||||||||||||||||||||||||||||||||||||||||||||||||||||||
Sbjct  704   CTGGCCCCTCCTCAGCATCTTATCCGAGTGGAAGGAAATTTGCGTGTGGAGTATTTGGAT  763

Query  719   GACAGAAACACTTTTCGACATAGTGTGGTGGTGCCCTATGAGCCGCCTGAGGTTGGCTCT  778
             ||||||||||||||||||||||||||||||||||||||||||||||||||||||||||||
Sbjct  764   GACAGAAACACTTTTCGACATAGTGTGGTGGTGCCCTATGAGCCGCCTGAGGTTGGCTCT  823

Query  779   GACTGTACCACCTTCCACTACAACTACATGTGTAACAGTTCCTGCATGGGCGGCATGAAC  838
             |||||||||||| |||||||||||||||||||||||||||||||||||||||||||||||
Sbjct  824   GACTGTACCACCATCCACTACAACTACATGTGTAACAGTTCCTGCATGGGCGGCATGAAC  883

Query  839   CGGAGGCCCATCCTCACCATCATCACACTGGAAAGACTCCAGTGGTAATCTACTGGGACG  898
             ||||||||||||||||||||||||||||||| ||||||||||||||||||||||||||||
Sbjct  884   CGGAGGCCCATCCTCACCATCATCACACTGG-AAGACTCCAGTGGTAATCTACTGGGACG  942

Query  899   GAACAGCTTTGAGGTGCGTGTTTGTGCCTGTCCTGGGAAGAGACCGGCGCACAGAGGAAG  958
             ||||||||||||||||||||||||||||||||||||| ||||||||||||||||||||||
Sbjct  943   GAACAGCTTTGAGGTGCGTGTTTGTGCCTGTCCTGGG-AGAGACCGGCGCACAGAGGAAG  1001

Query  959   AGAATCTCCGCAAGAAAGGGGAGCCTCACCACGAGCTGCCCCCAGGGAGCACTAAGCGAG  1018
             ||||||||||||||||||||||||||||||||||||||||||||||||||||||||||||
Sbjct  1002  AGAATCTCCGCAAGAAAGGGGAGCCTCACCACGAGCTGCCCCCAGGGAGCACTAAGCGAG  1061
```

```
Query  1019  CACTGCCCAACAACACCAGCTCCTYTCCCCAGCCAAAGAAGAAACCACTGG  1069
             ||||||||||||||||||||||| ||||||||||||||||||||||||||
Sbjct  1062  CACTGCCCAACAACACCAGCTCCTCTCCCCAGCCAAAGAAGAAACCACTGG  1112
```

We translate the RNA to protein (try to find out what kind of protein will come out.
Open following link:
https://www.ncbi.nlm.nih.gov/orffinder/
Copy and paste sequence to the empty yellow window.
Settings:
Genetic code: standard
ORF start (in case of p53) is ATG only.
Click on SUBMIT.
This program lists possible proteins, which can come out from the submitted mRNA sequence.
The longest possible protein coded by the submitted mRNA sequence will be listed first.
Using SMARTBLAST (click on SMARTBLAST) you can receive possible protein identifications.
Click on SMARTBLAST
The most similar (100 %) is the tumor suppressor protein p53 [Homo sapiens].

The result:

```
Query  1    MEEPQSDPSVEPPLSQETFSDLWKLLPENNVLSPLPSQAMDDLMLSPDDIEQWFTEDPGP
60
            MEEPQSDPSVEPPLSQETFSDLWKLLPENNVLSPLPSQAMDDLMLSPDDIEQWFTEDPGP
Sbjct  1    MEEPQSDPSVEPPLSQETFSDLWKLLPENNVLSPLPSQAMDDLMLSPDDIEQWFTEDPGP
60

Query  61   DEAPRMPEAAPRVAPAPAAPTPAAPAPAPSWPLSSSVPSQKTYQGSYGFRLGFLHSGTAK
120
            DEAPRMPEAAP VAPAPAAPTPAAPAPAPSWPLSSSVPSQKTYQGSYGFRLGFLHSGTAK
Sbjct  61   DEAPRMPEAAPPVAPAPAAPTPAAPAPAPSWPLSSSVPSQKTYQGSYGFRLGFLHSGTAK
120

Query  121  SVTCTYSPALNKMFCQLAKTCPVQLWVDSTPPPGTRVRAMAIYKQSQHMTEVVRRCPHHE
180
            SVTCTYSPALNKMFCQLAKTCPVQLWVDSTPPPGTRVRAMAIYKQSQHMTEVVRRCPHHE
Sbjct  121  SVTCTYSPALNKMFCQLAKTCPVQLWVDSTPPPGTRVRAMAIYKQSQHMTEVVRRCPHHE
180

Query  181  RCSDSDGLAPPQHLIRVEGNLRVEYLDDRNTFRHSVVVPYEPPEVGSDCTTFHYNYMCNS
240
```

```
                RCSDSDGLAPPQHLIRVEGNLRVEYLDDRNTFRHSVVVPYEPPEVGSDCTT HYNYMCNS
Sbjct  181     RCSDSDGLAPPQHLIRVEGNLRVEYLDDRNTFRHSVVVPYEPPEVGSDCTTIHYNYMCNS
240

Query  241     SCMGGMNRRPILTIITLE  258
               SCMGGMNRRPILTIITLE
Sbjct  241     SCMGGMNRRPILTIITLE  258
```

We have here, P72R AND I232F, I232F is not present in SCC-25 cells.

The patient has the I232F mutation in the DNA binding domain. This is a loss-of-function mutation. I232F is not detected in the TCGA project. (Ref: Mutational profiling can identify laryngeal dysplasia at risk of progression to invasive carcinoma - PMC (nih.gov))

3. **P53 sequence alignment of SCC-25 cells mixed cultured PD-CAFs cells with seq reference: 1126112 p53 standard**

```
Query  3     ACTTTGCGTTCGGGCTGGGAGCGTGCTTTCCACGACGGTGACACGCTTCCCTGGATTGGC  62
             ||||||||||||||||||||||||||||||||||||||||||||||||||||||||||||
Sbjct  56    ACTTTGCGTTCGGGCTGGGAGCGTGCTTTCCACGACGGTGACACGCTTCCCTGGATTGGC  115

Query  63    AGCCAGACTGCCTTCCGGGTCACTGCCATGGAGGAGCCGCAGTCAGATCCTAGCGTCGAG  122
             ||||||||||||||||||||||||||||||||||||||||||||||||||||||||||||
Sbjct  116   AGCCAGACTGCCTTCCGGGTCACTGCCATGGAGGAGCCGCAGTCAGATCCTAGCGTCGAG  175

Query  123   CCCCCTCTGAGTCAGGAAACATTTTCAGACCTATGGAAACTACTTCCTGAAAACAACGTT  182
             ||||||||||||||||||||||||||||||||||||||||||||||||||||||||||||
Sbjct  176   CCCCCTCTGAGTCAGGAAACATTTTCAGACCTATGGAAACTACTTCCTGAAAACAACGTT  235

Query  183   CTGTCCCCCTTGCCGTCCCAAGCAATGGATGATTTGATGCTGTCCCCGGACGATATTGAA  242
             ||||||||||||||||||||||||||||||||||||||||||||||||||||||||||||
Sbjct  236   CTGTCCCCCTTGCCGTCCCAAGCAATGGATGATTTGATGCTGTCCCCGGACGATATTGAA  295

Query  243   CAATGGTTCACTGAAGACCCAGGTCCAGATGAAGCTCCCAGAATGCCAGAGGCTGCTCCC  302
             ||||||||||||||||||||||||||||||||||||||||||||||||||||||||||||
Sbjct  296   CAATGGTTCACTGAAGACCCAGGTCCAGATGAAGCTCCCAGAATGCCAGAGGCTGCTCCC  355

Query  303   CGCGTGGCCCCTGCACCAGCAGCTCCTACACCGGCGGCCCCTGCACCAGCCCCCTCCTGG  362
             | ||||||||||||||||||||||||||||||||||||||||||||||||||||||||||
Sbjct  356   CCCGTGGCCCCTGCACCAGCAGCTCCTACACCGGCGGCCCCTGCACCAGCCCCCTCCTGG  415

Query  363   CCCCTGTCATCTTCTGTCCCTTCCCAGAAAACCTACCAGGGCAGCTACGGTTTCCGTCTG  422
```

```
             ||||||||||||||||||||||||||||||||||||||||||||||||||||||||||||
Sbjct  416   CCCCTGTCATCTTCTGTCCCTTCCCAGAAAACCTACCAGGGCAGCTACGGTTTCCGTCTG  475

Query  423   GGCTTCTTGCATTCTGGGACAGCCAAGTCTGTGACTTGCACGTACTCCCCTGCCCTCAAC  482
             ||||||||||||||||||||||||||||||||||||||||||||||||||||||||||||
Sbjct  476   GGCTTCTTGCATTCTGGGACAGCCAAGTCTGTGACTTGCACGTACTCCCCTGCCCTCAAC  535

Query  483   AAGATGTTTTGCCAACTGGCCAAGACCTGCCCTGTGCAGCTGTGGGTTGATTCCACACCC  542
             ||||||||||||||||||||||||||||||||||||||||||||||||||||||||||||
Sbjct  536   AAGATGTTTTGCCAACTGGCCAAGACCTGCCCTGTGCAGCTGTGGGTTGATTCCACACCC  595

Query  543   CCGCCCGGCACCCGCGTCCGCGCCATGGCCATCTACAAGCAGTCACAGCACATGACGGAG  602
             ||||||||||||||||||||||||||||||||||||||||||||||||||||||||||||
Sbjct  596   CCGCCCGGCACCCGCGTCCGCGCCATGGCCATCTACAAGCAGTCACAGCACATGACGGAG  655

Query  603   GTYGTGAGGCKCTGCCCCCWCCAWKASCRCTGCTCRGATAKCKATGGKCKGRCCCYTCCT  662
             || ||||||| |||||||| |||  | | |||||| |||| | |||| | | ||| ||||
Sbjct  656   GTTGTGAGGCGCTGCCCCCACCATGAGCGCTGCTCAGATAGCGATGGTCTGGCCCCTCCT  715

Query  663   CWGMMTCTTATCCTASTGGAWGGAGATTTGCGTGCGKAKKATTTGGATGACR-AAS-ACT  720
             | |  |||||||| | |||| ||| ||||||||| | |  |||||||||||  ||  |||
Sbjct  716   CAGCATCTTATCCGAGTGGAAGGAAATTTGCGTGTGGAGTATTTGGATGACAGAAACACT  775


Query  721   CTTCSASATAGTGTGKYGGYGMCCTATGAGYCGCCTGAGGTTGSYTCTGACTGTACCWSC  780
              ||| | ||||||||  || | |||||||| ||||||||||||  ||||||||||||  |
Sbjct  776   TTTCGACATAGTGTGGTGGTGCCCTATGAGCCGCCTGAGGTTGGCTCTGACTGTACCACC  835

Query  781   ATCCACTACAACTACATGTGTAACAGWTCCTGCATGASYGGCATGAACCGGAGGYSCATC  840
             |||||||||||||||||||||||||| |||||||||   |||||||||||||||  ||||
Sbjct  836   ATCCACTACAACTACATGTGTAACAGTTCCTGCATGGGCGGCATGAACCGGAGGCCCATC  895

Query  841   CTCACCATCATCACRCTGGAAGACTCSASTGGTAATCTACTGAGACGGAACAGCTTTGAG  900
             |||||||||||||| ||||||||||| | ||||||||||||| |||||||||||||||||
Sbjct  896   CTCACCATCATCACACTGGAAGACTCCAGTGGTAATCTACTGGGACGGAACAGCTTTGAG  955

Query  901   GTGMKYGTYTGTGCMTGTSCTGGGAKAGACCGGMGCWCASAGGAAGAGAATCTCCGCAAG  960
             |||   || ||||| ||| |||||| ||||||| || || ||||||||||||||||||||
Sbjct  956   GTGCGTGTTTGTGCCTGTCCTGGGAGAGACCGGCGCACAGAGGAAGAGAATCTCCGCAAG  1015

Query  961   AAATGGGAGCCTCACCACGAGCTGCTCCCAGGCAGCACTAAACGAGC  1007
             ||| ||||||||||||||||||||| |||||| |||||||| |||||
Sbjct  1016  AAAGGGGAGCCTCACCACGAGCTGCCCCCAGGGAGCACTAAGCGAGC  1062
```

Label areas that differ in Query and Subject, Start and End numbers are available.

We translate the RNA to protein (try to find out what kind of protein will come out.
Open following link:
https://www.ncbi.nlm.nih.gov/orffinder/
Copy and paste sequence to the empty yellow window.
Settings:
Genetic code: standard
ORF start (in case of p53) is ATG only.
Click on SUBMIT.
This program lists possible proteins, which can come out from the submitted mRNA sequence.
The longest possible protein coded by the submitted mRNA sequence will be listed first.
Using SMARTBLAST (click on SMARTBLAST) you can receive possible protein identifications.

1. Click on SMARTBLAST

```
2.  Query  1    MEEPQSDPSVEPPLSQETFSDLWKLLPENNVLSPLPSQAMDDLMLSPDDIEQWFTEDPGP  60
3.              MEEPQSDPSVEPPLSQETFSDLWKLLPENNVLSPLPSQAMDDLMLSPDDIEQWFTEDPGP
4.  Sbjct  1    MEEPQSDPSVEPPLSQETFSDLWKLLPENNVLSPLPSQAMDDLMLSPDDIEQWFTEDPGP  60
5.
6.  Query  61   DEAPRMPEAAPRVAPAPAAPTPAAPAPAPSWPLSSSVPSQKTYQGSYGFRLGFLHSGTAK  120
7.              DEAPRMPEAAP VAPAPAAPTPAAPAPAPSWPLSSSVPSQKTYQGSYGFRLGFLHSGTAK
8.  Sbjct  61   DEAPRMPEAAPPVAPAPAAPTPAAPAPAPSWPLSSSVPSQKTYQGSYGFRLGFLHSGTAK  120
9.
10. Query  121  SVTCTYSPALNKMFCQLAKTCPVQLWVDSTPPPGTRVRAMAIYKQSQHMTEVVRXCPXXX  180
11.             SVTCTYSPALNKMFCQLAKTCPVQLWVDSTPPPGTRVRAMAIYKQSQHMTEVVR CP
12. Sbjct  121  SVTCTYSPALNKMFCQLAKTCPVQLWVDSTPPPGTRVRAMAIYKQSQHMTEVVRRCPHHE  180
13.
14. Query  181  XCSDXXGXXXPXXLILXXGDLRAXXLDD  208
15.              CSD  G   P  LI   G+LR   LDD
16. Sbjct  181  RCSDSDGLAPPQHLIRVEGNLRVEYLDD  208
```

P72R is a neutral change. The yellow-labelled SCC-25-type deletion is present, whereas, the green-labelled patient specific p53 sequence change is not present. Additional changes are detected, which means that a mutator phenotype is activated in the SCC-25 cells cultured on CAFs. The CAFs might be still represented in the culture leading to heterozygous unclear sequences of p53.
