# Supplementary material for: Patient-Derived Cancer-Associated Fibroblasts Support the Colonization of Tumor Cells in Head and Neck Squamous Cell Carcinoma
Source: Biomedicines. 2025 Feb 4;13(2):358. doi: 10.3390/biomedicines13020358 (PMC11852712; doi:10.3390/biomedicines13020358)
Supplement: Supplementary file 1 [file biomedicines-13-00358-s001.zip › Supplementary Document S2.pdf]

Supplementary Document S2:

1. **FACS analysis of CAFs-educated SCC-25 cells.**

A)

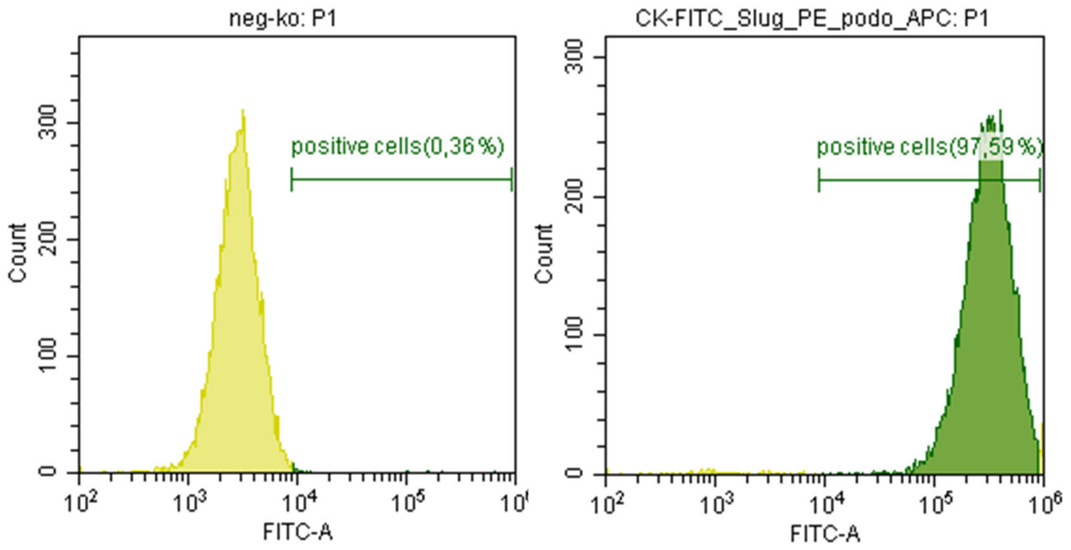

B)

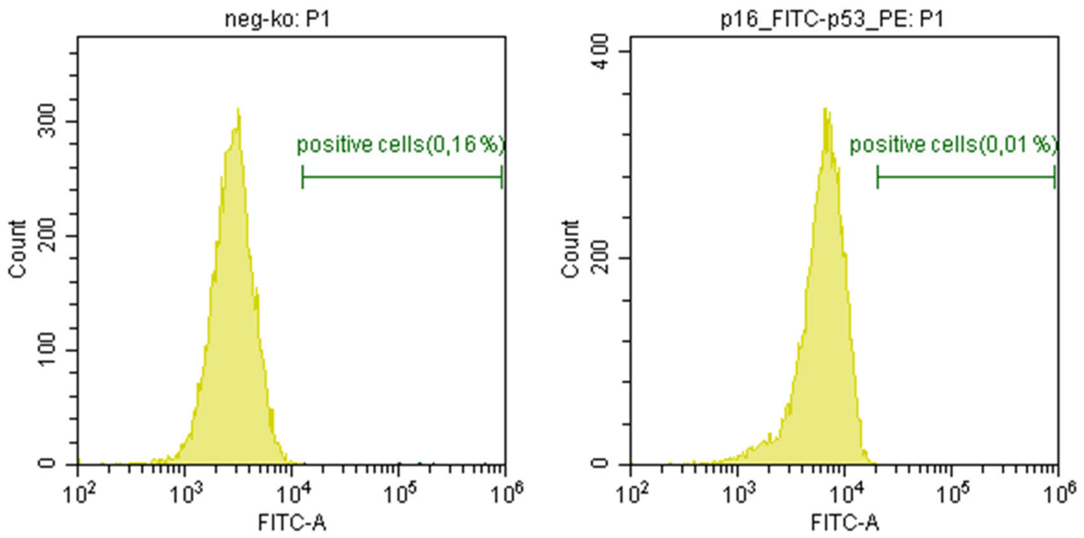

Figure S1: Passaged, CAFs-educated SCC-25 cells were subjected to FACS analysis and showed Cytokeratin positivity (A) and p16INK4 negative reaction (B).

2. **Zoomed subfigures of holotomographic images showing tumor cells in direct physical contact with CAFs. Extension of Figure 3.**

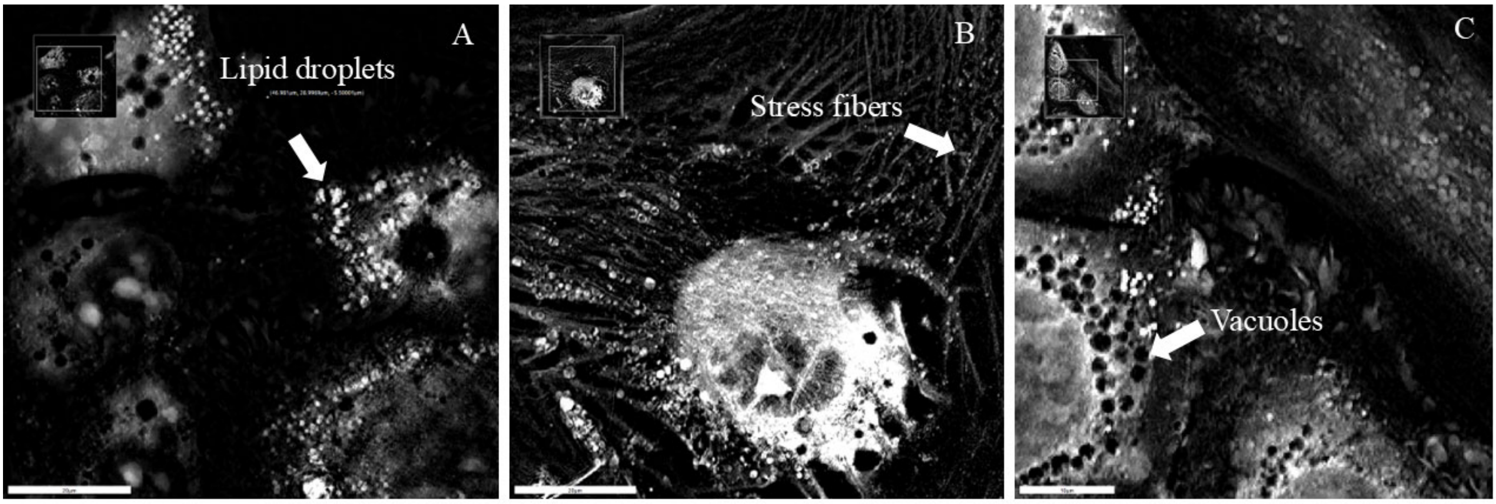

Figure S2: Mixed cultures of CAFs and SCC-25 cells were plated in 35 mm ibidi dishes. Morphological features and interactions between the CAFs and tumor cells were analyzed using a 3D Cell Explorer-fluo holotomographic microscope (air objective, 60× magnification). Imaging data were processed and analyzed using STEVE software. High-resolution subfigures presenting zoomed-in views of key observations. (A) Tumor cells exhibit lipid droplets at the cell edges (bright white dots) and vacuoles of varying sizes (black circles). (B) Co-cultured tumor cells and CAFs show stress fibers (straight-line structures) and signs of membrane disruption. (C) Direct interaction between tumor cells (left) and CAFs (right) reveals plasma membrane disruption in tumor cells, along with small vacuoles (black circles) within the tumor cells.
